# South African Medicinal Plants Traditionally Used for Wound Treatment: An Ethnobotanical Systematic Review

**DOI:** 10.3390/plants14050818

**Published:** 2025-03-05

**Authors:** Farzana Fisher (née Rahiman), Charlene Africa, Jeremy Klaasen, Randall Fisher

**Affiliations:** 1 Skin Research Lab, Department of Medical Biosciences, University of the Western Cape, Robert Sobukwe Rd, Bellville, Cape Town 7535, South Africa; klaasenjeremy89@gmail.com; 2 Maternal Endogenous Infections Studies, Department of Medical Biosciences, University of the Western Cape, Cape Town 7535, South Africa; cafrica@uwc.ac.za; 3 Separated Sector Cyclotron Lab, iThemba Laboratory for Accelerator-Based Sciences, Radiation Biophysics Division, Old Faure Rd, Eerste River, Cape Town 7100, South Africa; rg.fisher@ilabs.nrf.ac.za

**Keywords:** wound(s), skin, ethnobotany, survey, interview, traditional medicine, medicinal plants, South Africa

## Abstract

Microbial contamination of chronic wounds complicates their treatment. Traditional knowledge systems and the diversity of indigenous medicinal plants create a haven for traditional medicine practices in South Africa (SA). This systematic review aims to present a comprehensive ethnobotanical report of traditional medicines used in the documented empirical wound healing studies in SA. Google Scholar, PubMed, Medline EBSCOhost, Science Direct, and Scopus were sourced using the keywords/terminologies “South Africa”, “medicinal plants”, “traditional medicine” “indigenous”, “skin”, “wound”, “ethnobotany”, “survey”, “interview”, and “treatment” in different combinations. Relevant and unpublished records were retrieved from the Global Electronic Thesis Database. The searching process identified 32,419 records, of which 4005 studies were screened. Following the removal of 1795 duplicates, the remaining 2210 sources were screened by title and abstract, and 133 full-text reports were accessed and evaluated. Plants traditionally used for wound-healing purposes comprised 222 species belonging to 71 families, namely Asteraceae (predominantly the Helichrysum species), Asphodelaceae, Fabaceae, Solanaceae, and Euphorbiaceae. Plant organs used for medicinal remedies included leaves, roots, and bark prepared as poultices, infusions, decoctions, gel/ointments/lotions, and pastes. This review provides a valuable reference for future phytochemical and pharmacological studies and highlights the need for further ethnobotanical research to treat wounds in SA.

## 1. Introduction

Wounds can be defined as physical, chemical, or thermal injuries that disrupt the function, cellular, and anatomical integrity of the skin [1,2]. Globally, wounds have become a major health challenge affecting an estimated 14 million people, with 80% of cases occurring in developing countries [3]. The clinical and economic burden of wound care on the South African healthcare system remains unknown [4]. Lotz [5] investigated the burden of wound care in a resource-limited, tertiary hospital within the Western Cape Province and revealed that, in one day of the study, 34.6% of patients admitted presented with wounds and occupied 79% of vacant beds. These results concur with international reports associating billions of dollars with wound care costs [4,5].

Conventional medicine recommends antibiotic therapy for various skin conditions, including wounds. However, evidence suggests that microbial pathogens are continuously developing multidrug resistance, thereby compromising the effectiveness of this treatment approach and threatening public health [6,7,8]. Worldwide, South Africa (SA) has the highest prevalence of individuals living with the human immunodeficiency virus (HIV) and contributes to a third of the new HIV infections in the Southern part of Africa. Approximately 90% of persons infected with HIV develop a skin condition at some stage of the disease and have an increased susceptibility to wound infections as their immune systems weaken [9,10]. Consequently, most of these patients have a heightened incidence of bacterial infections and wound complications, in addition to a weakened and delayed wound-healing response [10]. Considering that skin conditions, including wounds, often persist among the immunocompromised and remain contagious for long periods, it is not surprising that patients are opting for alternate forms of therapy, such as traditional medicine, which is perceived to be better and safer for use [11].

The traditional use of plants as an alternate or adjunct form of therapy to Western medicine has largely become the backbone of rural healthcare in SA and the wider African continent [12,13]. SA has a mixture of cultures, beliefs, and languages that contribute to the diverse knowledge of traditional medicine [14]. Combined with the numerous plant species indigenous to SA, this makes the country a rich hub for traditional medicine practices [15]. For people residing in rural areas, there are several benefits associated with this form of treatment. This includes easy access to a traditional healer living close to their homes, the familiarity of a traditional healer with the individual’s cultural beliefs and surroundings and, importantly, the low cost associated with treatment [13]. Furthermore, herbal medicines are perceived to offer fewer side effects and better patient tolerance [16].

Agyare et al. [17] reported a selection of 61 southern African medicinal plant species with scientifically proven wound-healing properties. This list included the plant *Ageratum conyzoides*, used by traditional healers in SA for application to fresh wounds. Experiments using various plant extracts were assessed using excision, incision, and dead space wound models; the results revealed that methanol and aqueous plant extracts facilitated rapid wound healing. In a separate study, wounds treated with the bark extract of the plant species *Kigelia africana* showed improved collagenation, re-epithelisation, and granulation compared to untreated wounds in a rat excision model [18]. *Aspalathalus linearis*, commonly referred to as ‘Rooibos’, is a traditional herb used by the Khoisan in the form of a tea. Pringle et al. [19] conducted an in vitro assay that investigated the effect of fermented and aspathalin-enriched green rooibos on chronic wounds. Results showed that the pro-inflammatory activity of fermented rooibos had a beneficial effect on wounds, particularly wounds caused in the early stage of diabetes when the initial inflammatory response is delayed. On the other hand, green rooibos effectively protected fibroblasts against oxidative stress, suggesting a therapeutic potential for wounds with a heightened inflammatory response.

Ethnobotanical studies are largely used as a primary phase in identifying and developing pharmaceuticals from medicinal plants, as this could lead to the recognition of known and unknown biologically active compounds [20]. Studies of this nature are significant in identifying local plants used by communities for medicinal purposes [16]. Due to modernisation, valuable information from traditional healers on the wound-healing properties of plants has been lost, leaving a knowledge gap [2]. The need for ethnobotanical research to discover and record valuable medicinal knowledge cannot be over-emphasised [21].

A previous review by Mabona and Van Vuuren [11] examined the ethnobotanical literature and reported over 100 plant species traditionally used to treat skin conditions in southern parts of Africa, of which 41% were reportedly used for wounds. Another review assessing the available literature extending to the year 2017 identified only 20 plant species used historically to treat wounds in southern Africa [2]. Therefore, an in-depth compilation and analysis of the ethnobotanical studies reporting on wound treatment and management, specifically in SA and among certain cultural groups are warranted. This review aims to collate ethnobotanical reports from a comprehensive bibliographic search of empirical studies evaluating published and unpublished data on the traditional knowledge of plants used for wound care. The objectives are as follows: (a) to identify indigenous plants used in SA for wound healing; (b) to discuss traditional methods used in their preparation and application; and (c) to report on their biological properties. The outcome of this study will contribute to a global database of medicinally significant plant species, which will serve as a valuable reference for future phytochemical and pharmacological studies concerning remedies and ingredients for wound treatment.

## 2. Results and Discussion

### 2.1. Literature Search Results

**Figure 1:**
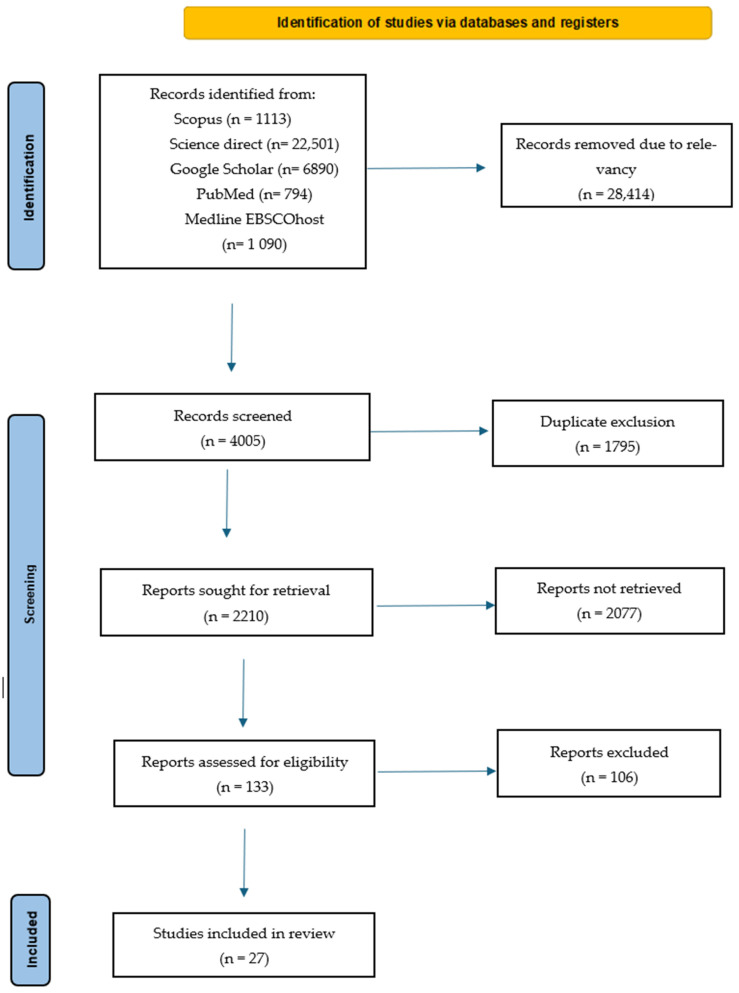
Workflow of selected sources of information.

A total of 32,419 studies were identified for preliminary review, of which 28,414 were considered irrelevant and excluded (Figure 1). The remaining 4005 records were screened, and 1795 duplicates were removed, leaving 2210 records for further evaluation. Using the specific inclusion and exclusion criteria, 133 articles and theses were assessed for eligibility and the final inventory for analysis and reporting comprised 27 papers.

### 2.2. Characteristics of the Ethnobotanical Studies Documenting the Use of Plants Traditionally Used to Treat Wounds in SA

This review reports on 27 ethnobotanical studies of medicinal plants traditionally used by South Africans to treat wounds (Table 1). As shown in Table 1, the studies were irregularly distributed over eight provinces in SA, with most of the research taking place in Limpopo (24%), the Eastern Cape (24%), and KwaZulu-Natal (17%). Other studies were accounted for in the provinces of the Northern Cape (10%), Western Cape (10%), North West (7%), and Mpumalanga (3%), with no relevant studies recorded in Gauteng. Limpopo is largely a rural province dominated by traditional medicine practices, owing to its rich plant diversity [12,22]. Despite numerous ethnobotanical studies exploring the medicinal value of plants in the province, there still seems to be a paucity of data concerning indigenous knowledge and practices on natural-based cosmeceuticals for skin conditions and wounds, specifically among certain ethnic groups, such as Vhavenda women [23]. This is corroborated by only seven studies conducted in the Limpopo region that report on plant usage for wound treatment. Considering the diverse and abundant flora within the Western Cape and North West provinces, and the large proportion of the South African population residing in rural areas who are highly dependent on traditional medicine, it is surprising that there is a shortage of ethnobotanical research investigating the increasing incidence of skin injuries, such as wounds [9,24,25]. However, other factors such as lack of awareness, inadequate funding, language, or cultural barriers, may have, contributed to the paucity of studies highlighted in this review [22,26]. The study conducted by Grierson and Afolayan [27] was the only report that intentionally focused on the use of medicinal plants for wound healing, while other studies covered topics related to general skin conditions or other ailments, thus revealing a gap in the research concerning wounds.

The included studies comprised sample sizes of 5–101 participants. Informants were classified as traditional health practitioners (healers, doctors, or sangomas), herbalists, indigenous knowledge holders, including medicinal plant sellers, village elders, and community members living in rural areas. The most common data collection method was structured or semi-structured interviews using questionnaires. Among the methods implemented were the Rapid Appraisal approach, the Matrix method, with the aid of a visual guide, or a combination of the aforementioned methodologies [28,29,30]. Such an approach to ethnobotanical research is advantageous, since this provides a means of becoming familiar with participants while obtaining insights that can be compared to the results of structured techniques [31]. Multi-disciplinary methods facilitate the collection of quantitative and qualitative data on contemporary theoretical problems concerning the development, nature, and transfer of ethnobotanical knowledge [22,31].

**Table 1:** Characteristics of the ethnobotanical studies on wound treatments in South Africa.

Study Reference	Region	Method of Data Collection	Number of Participants	Number of Plant Species	Number of Plant Families
Zwane et al., 2024 [32]	KwaZulu-Natal	Interview using a structured questionnaire	55	1	1
Ndhlovu et al., 2023 [33]	North West	Semi-structured interviews	101	4	4
Xaba et al., 2023 [34]	Free State	Interviews using structured questionnaires	10	17	13
Setshego et al., 2020 [35]	Limpopo	Semi-structured interviews	71	18	14
Asong et al., 2019 [9]	North West	Semi-structured questionnaires with a picture guide	30	13	9
Gebashe et al., 2019 [14]	KwaZulu-Natal	Interviews and questionnaires	60	1	1
Hulley and Van Wyk, 2019 [36]	Western Cape	Interviews using structured interviews	70	28	10
Mhlongo and Van Wyk, 2019 [29]	KwaZulu-Natal	Interviews using matrix method—picture guide	37	42	20
Mogale et al., 2019 [37]	Limpopo	Interviews	27	2	2
Ndhlovu et al., 2019 [23]	Limpopo	Interviews using semi-structured questionnaires	79	28	24
Thibane et al., 2019 [38]	Eastern Cape	Interviews using a structured questionnaire	50	5	5
Mongalo and Makhafola, 2018 [39]	Limpopo	Structured questionnaire and interviews.	40	2	2
Asowata-Ayodele et al., 2016 [40]	Eastern Cape	Interviews	74	1	1
Rankoana, 2016 [41]	Limpopo	Structured interviews	100	2	1
Tshikalange et al., 2016 [42]	Mpumalanga	Semi-structured interviews	15	6	5
Nortje and Van Wyk, 2015 [43]	Northern Cape	Semi-structured and structured interviews, questionnaires	24	20	12
Afolayan et al., 2014 [44]	Eastern Cape	Interviews and discussions	54	29	20
De Wet et al., 2013 [15]	KwaZulu-Natal	Interviews using a structured questionnaire	87	2	2
Josia, 2013 [8]	Eastern Cape	Interviews and questionnaires	37	31	17
Mahwasane et al., 2013 [13]	Limpopo	Interviews and questionnaires	30	1	1
Corrigan et al., 2011 [45]	KwaZulu-Natal	Interviews	5	4	4
De Beer and Van Wyk, 2011 [30]	Northern Cape	Interviews and questionnaires—Matrix method	16	7	5
Philander, 2011 [24]	Western Cape	Interviews	39	9	8
Van Wyk et al., 2008 [28]	Areas between Western Cape, Northern Cape, and Eastern Cape	Interviews (Rapid Appraisal approach)	7	6	6
Thring and Weitz, 2006 [46]	Western Cape	Interviews using questionnaires	44	4	4
Bhat and Jacobs, 1995 [47]	Eastern Cape	Surveys and interviews	Not reported	6	5
Grierson and Afolayan, 1999 [27]	Eastern Cape	General conversations with informants and questionnaires	Not reported	26	19

### 2.3. Medicinal Plants Identified for Wound Healing Purposes

**Figure 2:**
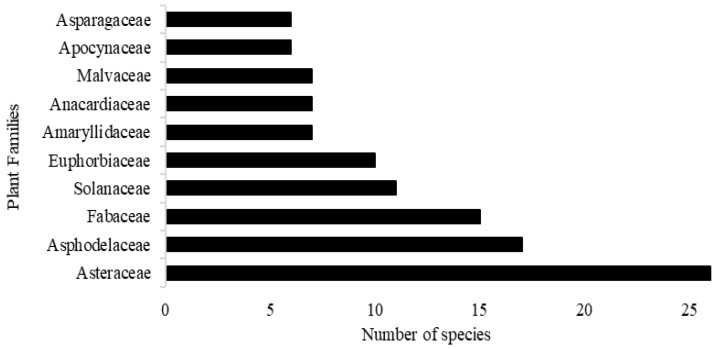
Ten traditionally used plant families with the highest number of species reported for wound healing.

This review collated information on the use of plants reported for wounds in general (63%) as well as wounds caused by cuts (3%), scrapes (1%), burns (24%), snake bites (6%), and circumcision (2%). A total of 222 species belonging to 71 families were identified as plants traditionally used for wound-healing purposes. Among all plant families reported, Asteraceae was the most cited (26 species), followed by Asphodelaceae (17 species), Fabaceae (15 species), Solanaceae (11 species), and Euphorbiaceae (10 species), respectively (Figure 2). Other families, some of which are indicated in Figure 2, were represented by 1–7 plant species.

### 2.4. Biological Activity of Commonly Identified Plant Families and Related Species

#### 2.4.1. Asteraceae

Asteraceaeous plants are widely distributed with over 1600 genera and 25,000 species globally. These plants inhabit sub-Saharan Africa, with 314 plants from 112 genera [33,48]. The extensive availability and abundance of various plant species may be the major reason for these plants being frequently cited for their medicinal use. Most Asteraceae family members have therapeutic applications with a well-established history of their use in traditional medicine [49]. They have been highly representative in numerous ethnobotanical studies, which may be linked to their phytochemical composition with associated medicinal significance [50]. Asteraceae plant species have demonstrated anti-inflammatory, antioxidant, antimicrobial, and wound-healing properties [48,49].

This review revealed the various *Helichrysum* species of the Asteraceae family as the plants predominantly used for wound healing. The *Helichrysum* species comprise essential oils and phytochemicals, such as phenolic acids, terpenes, flavonoids, and chalcones, which have often been associated with their therapeutic properties. As such, these plants are potential reservoirs of bioactive compounds for drug exploration and advancement. According to Akinyede et al. [51], only limited studies have been conducted on the biological effects of the *Helichrysum* species and their related phytochemicals.

Wound healing is a natural process, but complications can occur when a wound becomes infected with multidrug-resistant bacteria, thereby hindering the healing process and conventional treatment regimens. Plants possessing antimicrobial activity show promise as an option for wound repair and management [52]. Some *Helichrysum* species reported in this review, namely *Helichrysum petiolare* and *Helichrysum odoratissimum*, have demonstrated antimicrobial activity against gram-positive and gram-negative pathogens, supporting their traditional use in addressing wound infections [51,53]. Six different *Helichrysum* species, along with 21 other Asteraceae species, have been identified in the current review, of which only a few have been regularly reported for wound treatment. The frequent use of some of these plants could indicate that they are good candidates for further biochemical investigation [3]. The less reported plants should be further explored using in vitro and in vivo models. Such studies may enhance our current knowledge of the role of these plant species in wound healing, thereby creating a wider pool of plants for further development as cosmeceutical and/or pharmaceutical ingredients and products.

#### 2.4.2. Asphodelaceae

Data analysis showed two genera belonging to the Asphodelaceae family, namely *Aloe* (11 species) and *Bulbine* (6 species), as the most reported plants for wound care. Similar to Asteraceae, the probable reason for the increased usage of this family could be their extensive occupancy across the African continent. Aloes comprise about 548 accepted species, of which approximately 350 species are located in Africa, and are well recognised for their therapeutic properties [54]. Plants of the *Bulbine* genus are identified as succulent perennials, accounting for 78 species dispersed mainly in southern Africa, and widely used in traditional medicine [55]. *Aloe ferox* and *Aloe arborescens* (Table 2) have been used globally to treat and manage dermal wounds and burns.

*Aloe arborescens* is indigenous to South Africa and has been used by different cultural groups (Zulu, Xhosa, and Khoisan) to treat minor cuts, irritations, and burn wounds because of its anti-inflammatory activity [56]. Jia et al. [57] conducted a study investigating the wound-healing effect of *A. ferox* and *A. arborescens* on incisional wounds using a rat model and showed that both plants effected wound closure and facilitation of the healing process. Furthermore, these plants proved to have antimicrobial activity and no adverse effects on the skin, thereby supporting their use for dermatological application [57]. Other identified species of *Aloe*, namely *A. thraskii Baker* and *A. marlothii* have limited scientific evidence validating their wound healing effect, especially regarding their phytochemical and biological activity [58,59].

The traditional use of *Bulbine* species (Table 2) for its wound-healing properties is well established. Pather and Kramer [60] investigated the effect of *Bulbine natalensis* and *Bulbine frutescens* extracts on cutaneous wounds using a pig model. Results showed that wound closure increased significantly compared to the control group. Following treatment with leaf gels of the plants, the tensile strength of the wounds was observed to be much stronger, with an increase in protein, DNA, and collagen content [55,60]. Another study conducted by Hattingh et al. [61] included a scratch wound assay that showed an increase in the percentage of wound closure and migration rates of *B. frutescens* compared to control samples [61]. These recent studies scientifically validate the wound-healing properties of the *Bulbine* species. However, only a few species have been investigated, prompting questions related to the bioactivity of other lesser-known South African *Bulbine* species [4].

#### 2.4.3. Fabaceae

The Fabaceae family, commonly known as the legume or bean family, constitutes a group of medicinally and economically important plants, comprising 751 genera and an estimated 19,500 species [62,63]. The ethnobotanical studies conducted in the southern parts of Africa, including Namibia, Mozambique, and Zimbabwe have documented Fabaceae as a rich and therapeutic resource within these regions [64]. The frequent application of this plant species in traditional medicine is most likely linked to its wide distribution. Over the years, the sustained use of the Fabaceae plant family has been documented for its antibacterial, antifungal, antiviral, anticancer, anti-inflammatory, hepatoprotective, and neuroprotective effects. These biological activities have been associated with the plant’s phytochemical composition, revealing that this species contains crucial active metabolites [50,63].

Among the ten documented plant genera within the Fabaceae family, the *Desmodium*, *Erythrina*, and *Eriosema* species were cited for their therapeutic effect on wounds. In terms of wound repair, the antioxidant, anti-inflammatory, and antimicrobial activity of plant-derived bioactive compounds stimulate blood coagulation, combat infection, and accelerate the wound healing process [65]. A study performed by Pitkin et al. [66] aimed to establish the antimicrobial activity of *Desmodium incanum*, also referred to as the Beesbush plant. The well diffusion antimicrobial assay was employed and revealed that the Beesbush plant potently reduced the growth and replication of three microorganisms, namely *Staphylococcus aureus*, *Group D Streptococcus*, and *Klebsiella pneumoniae* at concentrations of 60 mg/dl and 100 mg/dl [66]. Considering that these species fall within a group of wound pathogens that exhibit multidrug resistance, further investigation into this plant species, as well as the rest of the genus, is warranted [67,68].

*Erythrina lysistemon*, *Erythrina caffra*, and *Erythrina latissimi* are plant species indigenous to South Africa, and their traditional application is reflective of their potential anti-inflammatory, antibacterial, and analgesic effects. Khumalo et al. [69] investigated the immunomodulatory effect of *E. lysistemon* to traditionally treat inflammatory conditions. Cytokine multiplex-bead assays were employed to assess the anti-inflammatory potential of the plant by evaluating the activity of interleukin-2 and interleukin-10 cytokines. Results showed that unstimulated murine RAW 264.7 macrophage cells exposed to the aqueous bark extract of *E. lysistemon* significantly upregulated levels of both anti-inflammatory cytokines with a 7000-fold increase in the level of IL-10 compared to the negative control [69]. The modulatory action of *E. lysistemon* is particularly noteworthy, and its effect may be linked to its inhibitory effect against natural killer cells, macrophages, and TH1 cells, which play an important role in tissue damage. In another study, the essential oil of *E. caffra* displayed antimicrobial activity against gram-positive and gram-negative bacteria. Furthermore, *E. caffra* showed the ability to scavenge superoxide and hydroxyl radicals, hydrogen peroxide, nitric monoxide, and singlet oxygen. This result is a good indicator of strong antioxidant activity, which is known to aid in the wound repair process [70]. 

To our knowledge, the ethnobotanical study by Mhlongo and Van Wyk [29] is the only one to have identified the two plant species *Eriosema cordatum* and *Eriosema distinctum* as plants traditionally used to heal wounds. Other reports on the traditional purposes of these plants include *E. cordatum*, used by Zulu traditional healers to address sexually related problems, such as erectile dysfunction or impotency, while *E. distinctum* has been reported to treat colds and influenza [71,72]. Several studies dealing with the antibacterial and antifungal effects of the *Eriosema* species have been documented; however, research focusing on the phytochemical, pharmacological, and toxicological evaluations of these two specific plants is limited [71], probably due to a lack of ethnobotanical studies identifying *E. cordatum* and *E. distinctum* for wound healing or other medicinal purposes.

#### 2.4.4. Amaryllidaceae, Aizoaceae, and Solanaceae

The plant species, *Boophane disticha*, *Carpobrotus edulis*, and *Datura stramonium*, belonging to the families Amaryllidaceae, Aizoaceae, and Solanaceae, respectively, were frequently cited (>5 studies) for wound healing purposes. In traditional medicine, *B. disticha* has been used for treating wounds, abdominal aches, and eye problems [73]. Phytochemical studies have revealed that crude extracts of *B. disticha* possess strong antioxidant activity, as reflected in their phenolic, alkaloid, and flavonoid content [74]. *C. edulis*, commonly referred to as the “Hottentot-fig” has been used as a herbal remedy for skin conditions such as burns, wounds, chilblains and eczema. The plant has been reported to be a rich source of bioactive compounds, associated with antioxidant, antimicrobial, and anti-inflammatory properties [75]. The biological activity of the plant species *D. stramonium* has also been demonstrated in vitro, highlighting its anti-inflammatory, antimicrobial, and antifungal activity potential. This evidence substantiates the traditional use of the plant in ayurvedic medicine to treat ulcers, wounds, bruises, and swelling [76].

### 2.5. Plant Parts Reported for Wounds

**Figure 3:**
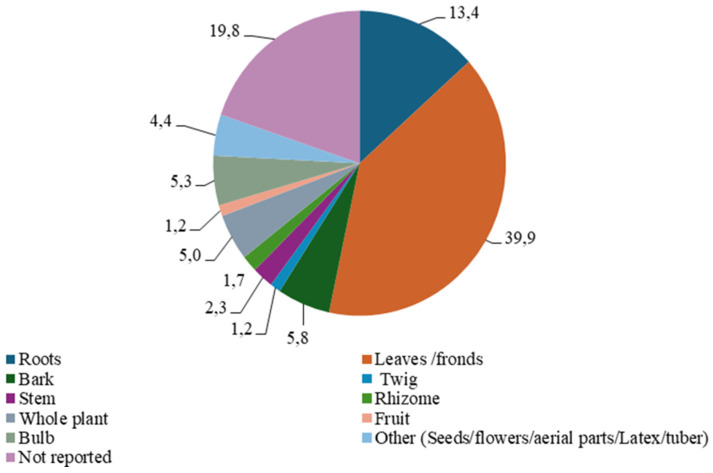
Parts of plants used to treat wounds.

The most cited plant organs used for medicinal remedies included leaves (39.9%) and roots (13.4 %), followed by the bark (5.8%); 21% of reports accounted for other plant parts (Figure 3). This result is supported by the predominant use of leaves for treating skin conditions documented in various ethnobotanical studies [44,77,78,79,80,81]. According to Aumeeruddy and Mahomoodally [82], the high usage of leaves is a general trend observed in traditional medicine and could be attributed to their abundance and the ease with which they are obtained compared to other plant parts. Leaves are the primary photosynthetic organ of plants and are regarded as key components of phytoconstituents. The preference for leaves may specifically be due to their high quantity of active ingredients like tannins and alkaloids [83]. The high frequency of roots used in herbal remedies is also believed to be related to their phytochemical composition and heightened pharmacological activity, compared to other plant parts [83,84]. It is important to note that approximately 20% of the studies did not provide information on the plant parts used. Anecdotal reports suggest that some traditional healers are not entirely comfortable sharing in-depth knowledge with researchers due to a lack of trust or fear of product development without any acknowledgement or benefit.

### 2.6. Methods of Preparation and Route of Administration

**Figure 4:**
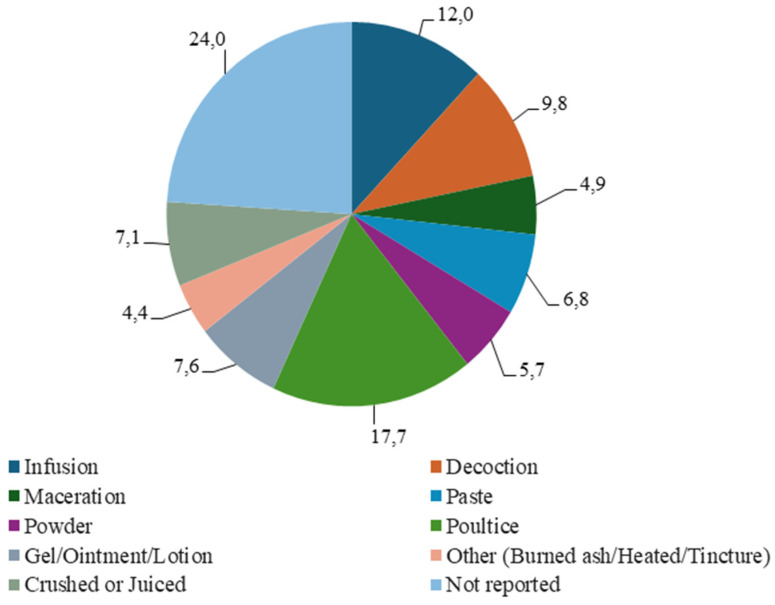
Mode of preparation for plants used to treat wounds.

The major methods of preparation recorded were poultices (17.7%) and infusions (12%). This was followed by decoctions (9.8%), gels/ointments/lotions (7.6%), and pastes (6.8%) (Figure 4). A lack of reporting by authors (24%) was once again observed on this aspect of the included studies. The formulation of herbal medicines is a crucial process, since it determines how the therapeutic properties of the plant are best released to effectively treat a condition [85]. The increased use of poultices for wounds can be attributed to this being a simple method of application to a wound or body part. This result is similar to other studies conducted in countries such as Malaysia and Uganda, where poultices are the most popular method for wound treatment [3]. Reports have indicated that well-known plants for wound used for healing purposes, namely *Ageratum conyzoides*, *Althaea officinalis*, and *Symphytum officinale* are prepared in the form of poultices because this preparation method aids in speedy recovery and reduces bacterial infection [86]. In SA, infusion is often used as a mode of preparation for herbal remedies and is considered an effective process, since only a few resources are needed. The process is uncomplicated and entails combining plant material with boiled water for a certain period [87]. Evidence suggests that bioactive compounds contained in herbal infusions may be linked to a diverse range of effects inclusive of anti-inflammatory, antioxidant, and antibacterial properties [88], all of which play a significant role in facilitating the process of wound repair.

**Figure 5:**
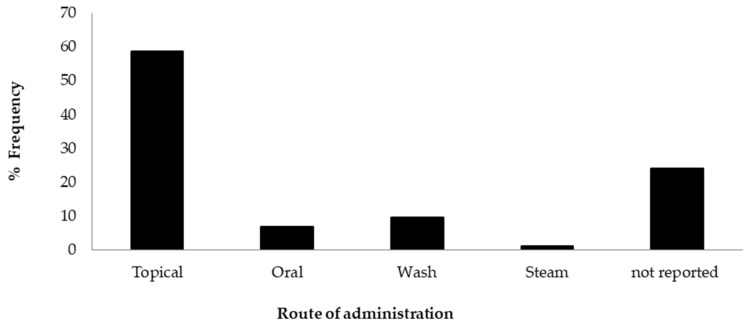
Administration routes for medicinal plants used to treat wounds.

Figure 5 illustrates the dominance of topical applications as the popular route of administration for herbal remedies (59%). This correlates with the high reports of poultices used in the treatment of wounds as well as other topical treatments, such as the use of lotions, gels, powders, or pastes, documented in the included studies. According to Gwarzo et al. [89], the topical application of a cream creates a moist environment that prevents dryness of the affected skin and enhances the repair process by mitigating inflammation and preventing colonisation of the wound by microbial pathogens. This is in contrast to reports from West Africa, where community members believe that keeping a wound dry by applying sand, ash, or herbal remedies promotes wound healing [90].

Furthermore, topical application has a lower potential for absorption and toxicity [91]. Other modes of delivery, such as oral, wash, and steam, had lower frequency reports, within the range of 1–9%, while a significant number of the studies lacked reporting on this characteristic. It is particularly interesting that more than one route of administration was used. According to Alamgeer et al. [92], topical application is still a preferred and effective approach, as it allows direct contact of active constituents of the plant with the skin, providing rapid relief [92,93].

### 2.7. Conservation Status

Environmental conservation is a crucial source of income for local communities and, in sub-Saharan Africa, efforts towards the sustainable use of medicinal plants have been well emphasised [87]. Traditional healers have stringent cultural beliefs concerning the cultivation of medicinal plants and have therefore supported the conservation of these plants. Some of the traditional practices that have prevented plant species from being exploited include harvesting plant parts following the approval of ancestors and specific rituals, collection of only two roots of the same plant at one point in time and harvesting specific plants during winter to ensure the seed set and growth during the summer season. However, the shift in harvesting plant material from subsistence to commercial traders has contributed to the lower likelihood of traditional healers implementing these practices [94].

An assessment of the conservation status of various identified plants was conducted according to the SANBI Red List of South African Plants (http://redlist.sanbi.org, accessed on 15 October 2024). Results revealed that 77% of the species were of least concern (LC), 9% were invasive alien species, and 10% had not been evaluated. Four plants were classified as endangered/near threatened (*Merwilla plumbea* (Lindl.) *Speta*, *Eucomis bicolor* Baker, *Aloe thraskii* Baker, and *Alepidea amatymbica*), three plants (*Brackenridgea zanguebarica* Oliv., *Euphorbia bupleurifolia* Jacq., and *Kniphofia drepanophylla* Baker) were identified as vulnerable (critically endangered) and one species (*Aptosimum procumbens* (Lehm.) Burch. ex Steud.) was categorised as rare. The preferred use of leaves reported in this paper suggests traditional harvesting methods are indicative of a sustainable approach aimed at protecting plant species. Although most plants are of least conservation concern, the parts harvested from plants within the other categories included the use of bulbs, roots, rhizomes, or the entire plant. Using these plant parts can have a detrimental effect on the reproduction of medicinal plants in an area [92]. Furthermore, the harvesting of roots, stems, and bark has damaging effects on a plant, resulting in low heterogeneity and richness of a plant species [26]. Factors such as unplanned collection of plant material, increased exploitation, extensive grazing, prepping of land for deforestation, agriculture and erosion, and the attack of pathogens, all pose a significant threat to medicinal plants [92]. It is increasingly important to protect the future of plants considering their increased susceptibility to population decline when harvested intensively in an unsustainable way [87]. South Africa has recognised this issue and has implemented various strategies supported by provincial governments and municipalities to ensure the effective propagation processes for threatened plant species and to assess the effect of cultivation on the biological activity of plants with therapeutic benefits [94].

## 3. Materials and Methods

This review was conducted according to the Preferred Reporting Items for Systematic Reviews and Meta-Analyses (PRISMA) guidelines [95] and has not been registered. Electronic databases, namely Google Scholar, PubMed, Medline EBSCOhost, Science Direct, and Scopus, were sources for this review. The Global Electronic Thesis Database (http://search.ndltd.org, accessed on 10 August 2024) was further examined to access all relevant and unpublished records. The search strategy had no limitation in terms of publication status or language. All records up to and including July 2024 were searched. Primary search keywords or terminologies, such as “South Africa”, “medicinal plants”, “traditional medicine” “indigenous”, “skin”, “wound”, “ethnobotany”, “survey”, “interview”, and “treatment”, were combined using Boolean operators, with at least three key words in a single search. The studies were evaluated based on the following selection criteria: (a) Only primary ethnobotanical studies reporting on medicinal plants used to treat wounds in SA were included. (b) All lab-based studies, clinical trials, review articles, books, editor’s comments, and letters were excluded. To reduce bias in the selection and collection of data, the authors, along with the assistance of five student researchers, first worked independently and then together during the search (January 2022 to July 2024) and data collection (August 2024 to October 2024) processes. Any misinterpretations or disagreements were resolved when a consensus was reached based on a discussion between the researchers.

The ethnobotanical information pertaining to the scientific name of plant species, method of preparation, part of the plant used, and route of administration were extracted from the included sources. In the case of a study reporting on other types of skin-related conditions, only the relevant information on wounds was extracted. For studies including various methodologies, only the data related to the ethnobotanical research were extracted. Knowledge obtained directly from participants in the studies was included in this review and information on traditional applications of plants cited from the previous literature was not considered. The accuracy of plant names was confirmed using the Plant List (https://wfoplantlist.org, accessed on 20 February 2025) and SANBI websites (http://pza.sanbi.org, accessed on 25 August 2024; http://redlist.sanbi.org accessed 30 September 2024). These platforms were accessed regularly during the data extraction period. As such, unidentified plants reported in the studies were omitted from the data analysis. Descriptive statistics was performed using Microsoft Excel to present graphs and figures relevant to the results retrieved.

## 4. Conclusions

This study provides evidence of the remedies and ingredients traditionally used for wound treatment from 27 ethnobotanical studies. Because much of the indigenous knowledge of the use of traditional medicines has been lost and some plant species face extinction if not appropriately propagated and protected, South Africa has implemented strategies to protect its vast array of medicinal plants to ensure their sustainability for the future. Further reporting of plant species not covered in the previous studies is warranted.

The use of plants as an alternate treatment strategy has become increasingly important given the lack of access to appropriate antibiotics, which is a key factor in driving antimicrobial resistance [96]. The ability of infecting microbes to form biofilms, combined with the rapid increase in antimicrobial resistance, hampers the effective treatment of wounds. Most pharmaceutical antimicrobials do not penetrate microbial biofilms efficiently, creating a need for new strategies for wound management. Considering the antimicrobial potential of various plant-based products, the ability of plants to prevent biofilm formation, while also promoting tissue healing is worth further exploration [97].

Composed of several bioactive compounds with anti-inflammatory, antimicrobial, and healing properties [98,99], medicinal plants have, over time, developed mechanisms to identify and defend against invading pathogens [99,100]. Exploring their different mechanisms, bioavailability, and properties to develop new antimicrobials with low minimal inhibitory concentrations will contribute significantly to the search for novel, low-cost alternative therapies to address the problem of antimicrobial resistance, while simultaneously preserving mammalian cells and complementing the existing indigenous knowledge.

Globally, the efficacy of plants with wound-healing properties has been well established and various patented plant formulations have led to commercialisation [101,102]. Aloe vera is an example of a plant that has undergone clinical trials proving its wound-healing capability [103], thereby leading to product development. Most botanicals identified in this review have demonstrated beneficial properties in vitro [65,69,70,71,74,75,76]. However, their clinical investigation is limited. As with the assessment of any drug, these medicinal plants warrant clinical trials to fully comprehend their therapeutic benefits and to determine any possible side effects, such as allergic reactions.

A limitation of this review is that the evidence presented comprised studies with a significant percentage of missing data for the outcomes assessed. Information provided by the participants may have differed from those that did not report, thereby limiting this review in providing a comprehensive report of the plants used to treat wounds in South Africa. Nevertheless, this paper presents valuable results adding to a limited pool of information and contributes to a global database of South African plant species with wound-healing properties.

## Figures and Tables

**Table 2:** Plants traditionally used for wound healing in South Africa.

	Family	Scientific Name of Plant Species	Method of Preparation	Plant Parts Used	Route of Administration	References
1	Asteraceae	*Acanthospermum hispidum* DC.	Maceration	Leaves	Topical	[23]
2	Asteraceae	*Acmella caulirhiza* Delile	Crushed	Leaves	Topical, wash	[35]
3	Apocynaceae	*Acokanthera oppositifolia* (Lam.) Codd	Paste Not reported Decoction	Leaves, stem Not reported Leaves	Topical Not reported Topical	[29,44,47]
4	Lamiaceae	*Acrotome inflata* Benth.	Not reported	Rhizome	Not reported	[9]
5	Malvaceae	*Adansonia digitata* L.	Maceration	Bark	Topical	[23]
6	Asteraceae	*Afroaster hispidus* (Thunb.) J.C. Manning & Goldblatt	Not reported	Not reported	Not reported	[29]
7	Fabaceae	*Afzelia quanzensis* Welw	Infusion	Bark, root	Oral	[34]
8	Rosaceae	*Agrimonia eupatoria* L.	Decoction	Leaves	Topical	[44]
9	Hyacinthaceae	*Albuca bracteata* (Thunb.) J.C. Manning & Goldblatt	Not reported	Not reported	Not reported	[29]
10	Hyacinthaceae	*Albuca setosa* Jacq.	Paste	Leaves	Topical	[44]
11	Hyacinthaceae	*Albuca virens* (Lindl.) J.C. Manning & Goldblatt subsp. virens	Not reported	Not reported	Not reported	[29]
12	Apiaceae	*Alepidea amatymbica* Eckl. & Zeyh.	Maceration, decoction Infusion	Roots, rhizome Roots	Oral, topical Not reported	[34] [40]
13	Asphodelaceae	*Aloe aageodonta* L.E.Newton	Poultice, paste, gel	Leaves	Topical	[23]
14	Asphodelaceae	*Aloe arborescens* Mill.	Paste, gel Not reported	Leaves Not reported	Topical Not reported	[8] [29]
15	Asphodelaceae	*Aloiampelos striatula* (Haw.)	Poultice	Leaves	Topical	[27]
16	Asphodelaceae	*Aristaloe aristata* (Haw.) Boatwr. & J.C.Manning	Infusion	Leaves	Wash	[34]
17	Asphodelaceae	*Aloe vera* (L.) Burm. f.	Sap	Leaves	Topical	[35]
18	Asphodelaceae	*Aloe ferox* Mill.	Not reported Gel Not reported Decoction Not reported Juice Not reported	Not reported Leaves Leaves Leaves Not reported Leaves Not reported	Not reported Topical Topical Oral Not reported Topical Not reported	[29] [8] [44] [34] [43] [27] [36]
19	Asphodelaceae	*Aloe maculata* All.	Not reported Infusion, maceration	Not reported Leaves, stem, and rhizome	Not reported Oral, topical	[29] [33]
20	Asphodelaceae	*Aloe marlothii* A. Berger	Poultice Infusion	Leaves Leaves	Topical Topical	[23] [39]
21	Asphodelaceae	*Aloe microstigma* Salm-Dyck	Juice Heated	Leaves Not reported	Topical, wash Topical	[43] [30]
22	Asphodelaceae	*Aloe thraskii* Baker	Not reported	Not reported	Not reported	[29]
23	Anacampserotaceae	*Anacampseros papyracea* E. Mey. ex Fenzl subsp. *papyracea*	Not reported	Not reported	Not reported	[36]
24	Commelinaceae	*Aneilema aequinoctiale* (P.Beauv.) Loudon	Not reported	Not reported	Not reported	[29]
25	Scrophulariaceae	*Aptosimum indivisum* Burch. ex Benth.	Powdered	Leaves	Topical	[36]
26	Scrophulariaceae	*Aptosimum procumbens* (Lehm.) Burch. ex Steud.	Powdered ash Not reported Not reported	Whole plant Foliage Not reported	Topical Topical Not reported	[28] [24] [36]
27	Asteraceae	*Arctotis arctotoides* (L.f) O.Hoffm	Paste Juice	Leaves Leaves	Topical Topical	[38] [44]
28	Asteraceae	*Artemisia afra* Jacq. ex. Willd.	Infusion	Leaves	Wash	[27]
29	Asparagaceae	*Asparagus africanus* Lam.	Infusion Decoction	Leaves Roots	Topical Oral	[44] [34]
30	Asteraceae	*Athrixia phylicoides* DC.	Crushing	Leaf twigs, roots	Topical	[8]
31	Iridaceae	*Babiana hypogaea* Burch	Not reported	Leaves	Not reported	[9]
32	Lamiaceae	*Ballota africana* (L.) Benth.	Infusion Infusion	Leaves Leaves	Topical, wash Wash	[43] [36]
33	Acanthaceae	*Barleria obtusa* Nees	Paste	Leaves	Topical	[44]
34	Acanthaceae	*Barleria* sp.	Not reported	Roots	Not reported	[9]
35	Acanthaceae	*Barleria macrostegia* Nees	Not reported	Roots	Not reported	[9]
36	Fabaceae	*Bauhinia thonningii* (Schumach.) Milne-Redh.	Juice	Fruit	Topical	[35]
37	Apiaceae	*Berula erecta* (Huds.) Coville subsp. *thunbergii* (DC.)	Not reported	whole plant	Topical	[28]
38	Asteraceae	*Bidens pilosa* L.	Poultice Crushed Not reported	Leaves Leaves Not reported	Topical Topical Not reported	[23] [35] [29]
39	Amaryllidaceae	*Boophone disticha* (L.f.) Herb.	Crushing Paste Not reported Not reported Poultice Poultice	Bulb and leaves Bulb Bulb Dry bulb Bulb and leaves Bulb and leaves	Topical Topical Topical Topical Topical Topical	[8] [27] [24] [28] [37] [36]
40	Asteraceae	*Brachylaena discolor* DC.	Infusion	Roots	Topical	[44]
41	Ochnaceae	*Brackenridgea zanguebarica* Oliv.	Maceration	Bark	Topical	[23]
42	Amaryllidaceae	*Brunsvigia grandiflora* Lindl.	Crushed	Leaves, Bulb	Topical	[47]
43	Asphodelaceae	*Bulbine asphodeloides* (L.) Spreng.	Paste, gel Not reported	Leaves Not reported	Topical Not reported	[8] [29]
44	Asphodelaceae	*Bulbine foleyi* E. Phillips	Not reported	Foliage	Topical	[24]
45	Asphodelaceae	*Bulbine frutescens* (L.) Willd.	Juice, gel Juice Juice Juice	Leaves Leaves Leaves Leaves	Topical Topical Not reported Topical	[8] [38] [43] [36]
46	Asphodelaceae	*Bulbine lagopus* (Thunb.) N.E.Br.	Juice	Leaves	Topical	[46]
47	Asphodelaceae	*Bulbine latifolia* (L.f.) Spreng. (=*Bulbine natalensis* Baker)	Poultice Not reported	Leaves Not reported	Topical Not reported	[27] [29]
48	Cannaceae	*Canna indica* L.	Poultice	Leaves	Topical	[36]
49	Capparaceae	*Capparis tomentosa* Lam.	Powder	Roots	Topical	[44]
50	Apocynaceae	*Carissa edulis* (Forssk.) Vahl.	Infusion	Leaves	Wash	[39]
51	Aizoaceae	*Carpobrotus edulis* (L.) L.Bolus subsp. *Parviflorus* Wisura & Glen.	Lotion Not reported Juice Not reported Not reported Not reported	Leaves Leaves Leaves Foliage Not reported Not reported	Oral, topical Topical Topical Topical Not reported Topical	[34] [44] [8] [24] [43] [30]
52	Apiaceae	*Centella asiatica* (L.) Urb	Not reported Not reported Tinctures Poultice, lotion	Whole plant Not reported Leaves Leaves	Not reported Not reported Topical Topical	[9] [29] [44] [27]
53	Asteraceae	*Centaurea benedicta* (L.) L.	Infusion	Whole plant	Oral	[8]
54	Asteraceae	*Centaurea scabiosa* L.	Infusion	Whole plant	Topical	[33]
55	Asteraceae	*Vernonia* spp.	Lotion	Whole plant	Topical	[42]
56	Celtidaceae	*Chaetachme aristata* Planch.	Burned ash	Bark	Topical	[45]
57	Pteridaceae	*Cheilanthes viridis* (Forssk.) Sw. var. viridis	Powder	Fronds	Topical	[27]
58	Gentianaceae	*Chironia baccifera* L.	Infusion	Whole plant	Not reported	[36]
59	Menispermaceae	*Cissampelos capensis* L.f.	Poultice	Leaves	Topical	[36]
60	Euphorbiaceae	*Clutia ovalis* Sond.	Not reported	Not reported	Not reported	[29]
61	Nyctaginaceae	*Commicarpus pentandrus* (Burch) Heimerl	Not reported	Whole plant	Not reported	[9]
62	Burseraceae	*Commiphora mollis* (Oliv.) Engl.	Maceration	Bark	Topical	[23]
63	Burseraceae	*Commiphora harveyi* (Engl.) Engl.	Not reported	Not reported	Not reported	[29]
64	Crassulaceae	*Cotyledon orbiculata* L.	Poultice Ointment Poultice	Leaves and cuticle Leaves Leaves	Topical Topical Topical	[27] [30] [43]
65	Amaryllidaceae	*Crossyne guttata* (L.) D.Müll.-Doblies & U.Müll.-Doblies	Not reported	Bulb	Topical	[24]
66	Poaceae	*Cynodon dactylon* (L.) Pers.	Burnt, ash powder	Whole plant	Topical	[14]
67	Fumariaceae	*Cysticapnos vesicaria* (L.) Fedde subsp. vesicaria	Not reported	Not reported	Wash	[36]
68	Amaryllidaceae	*Cyrtanthus obliquus* (L.f.) Aiton	Burned Ash	Dry or fresh roots	Topical	[47]
69	Thymelaeaceae	*Dais cotinifolia* L.	Not reported	Not reported	Not reported	[29]
70	Solanaceae	*Datura stramonium* L.	Heating Heating Paste Poultice, ointment Poultice	Leaves Leaves Seeds Leaves, seeds Leaves	Topical Topical Topical Topical Topical	[44] [8] [37] [43] [36]
71	Fabaceae	*Desmodium incanum* (Sw.) DC.	Not reported	Not reported	Not reported	[29]
72	Fabaceae	*Desmodium setigerum* (E.Mey.) Benth. ex Harv.	Not reported	Not reported	Not reported	[29]
73	Fabaceae	*Dichrostachys cinerea* (L.) Wight & Arn	Poultice Burned, decoction Decoction, infusion	Leaves Fruit, Bark Roots, Pods	Topical Topical, wash Not reported	[23] [35] [42]
74	Asteraceae	*Dicoma anomala* Sond.	Not reported	Roots	Not reported	[9]
75	Dioscoreaceae	*Dioscorea elephantipes* (L’Her.) Engl.	Infusion, Decoction, Lotion	Whole plant	Topical	[34]
76	Dioscoreaceae	*Dioscorea sylvatica* Eckl.	Infusion	Tuber	Topical	[34]
77	Ebenaceae	*Diospyros lycioides* Desf.	Juice	Fruit	Wash	[35]
78	Asparagaceae	*Drimia* species	Poultice	Bulb scales	Topical	[46]
79	Boraginaceae	*Ehretia rigida* (Thunb.) Druce	Poultice	Leaves	Topical	[23]
80	Meliaceae	*Ekebergia capensis* Sparrm.	Maceration, poultice	Leaves	Topical	[23]
81	Fabaceae	*Elephantorrhiza elephantina* (Burch) Skeels.	Maceration, poultice Decoction, ointment	Roots Roots	Oral, topical Oral, topical	[33] [34]
82	Asteraceae	*Elytropappus rhinocerotis* (L.f.) Less.	Infusion Burned ash	Leaves Aerial parts	Wash Not reported	[46] [36]
83	Musaceae	*Ensete ventricosum* (Welw.) E.E. Cheesman	Poultice, lotion	Leaves	Topical	[23]
84	Fabaceae	*Eriosema cordatum* E.Mey.	Not reported	Not reported	Not reported	[29]
85	Fabaceae	*Eriosema distinctum* N.E.Br.	Not reported	Not reported	Not reported	[29]
86	Fabaceae	*Erythrina caffra* Thunb.	Powder	Bark	Topical	[27]
87	Fabaceae	*Erythrina latissima* E.Mey.	Not reported	Not reported	Not reported	[29]
88	Fabaceae	*Erythrina lysistemon* Hutch.	Powder, Decoction	Bark	Topical, Oral	[44]
89	Myrtaceae	*Eucalyptus globulus* Labill. subsp. *maidenii* (F.Muell.) J.B.Kirkp.	Not reported	Foliage	Wash	[24]
90	Ebenaceae	*Euclea divinorum* Hiern	Poultice	Leaves	Topical	[23]
91	Asparagaceae	*Eucomis autumnalis* (Mill). Chitt	Decoction Not reported Crushed	Bulbs, roots Not reported Bulb	Oral Not reported Topical	[34] [29] [8]
92	Asparagaceae	*Eucomis bicolor* Baker.	Decoctions, infusion	Bulbs	Oral	[34]
93	Myrtaceae	*Eugenia capensis* subsp. *natalitia* (Sond.) F.White	Maceration (Bark) Infusion (Roots)	Bark, roots	Topical, wash	[23]
94	Euphorbiaceae	*Euphorbia bupleurifolia* Jacq.	Not reported	Latex	Topical	[44]
95	Euphorbiaceae	*Euphorbia cupularis* Boiss.	Not reported	Not reported	Not reported	[29]
96	Euphorbiaceae	*Euphorbia inaequilatera* Sond.	Not reported	Roots	Not reported	[9]
97	Euphorbiaceae	*Euphorbia tirucalli* L.	Not reported	Leaves	Not reported	[41]
98	Aizoaceae	*Galenia africana* L.	Decoction Infusion, ointment	Not reported Leaves, twigs	Wash Wash, topical	[28] [43]
99	Asteraceae	*Gerbera piloselloides* (L.) Cass.	Not reported Infusion	Not reported Roots	Not reported Topical	[29] [8]
100	Colchicaceae	*Gloriosa superba* L.	Not reported	Not reported	Not reported	[29]
101	Thymelaeaceae	*Gnidia anthylloides* (L.f.) Gilg	Burnt and crushed	Roots	Topical	[8]
102	Thymelaeaceae	*Gnidia capitata* (L.f.) Burtt Davy	Decoction, burnt, and crushed	Roots	Topical, wash	[8]
103	Thymelaeaceae	*Gnidia kraussiana* Meisn.	Not reported	Leaves	Topical	[44]
104	Apocynaceae	*Gomphocarpus physocarpus* E.Mey.	Not reported	Not reported	Not reported	[29]
105	Apocynaceae	*Gomphocarpus fruticosus* (L) Aiton.f.	Not reported	Whole plant	Not reported	[9]
106	Asphodelaceae	*Gonialoe variegata* (L.) Boatwr. & J.C.Manning (=Aloe variegata L.)	Poultice Poultice Poultice Not reported	Leaves Leaves Leaves Not reported	Topical Topical Topical Not reported	[43] [36] [30] [28]
107	Malvaceae	*Grewia occidentalis* L.	Infusion, lotion	Small twigs and leaves	Topical	[27]
108	Gunneraceae	*Gunnera perpensa* L.	Poultice Decoction	Leaves Rhizomes	Topical Oral	[27] [8]
109	Celastraceae	*Gymnosporia buxifolia* (L.) Szyszyl.	Infusion	Leaves, Roots	Not reported	[42]
110	Celastraceae	*Gymnosporia rubra* (Harv.) Loes.	Not reported	Not reported	Not reported	[29]
111	Amaryllidaceae	*Haemanthus albiflos* Jacq.	Paste Infusion Decoction	Leaves Roots Bulb	Topical Oral Oral	[44] [34] [38]
112	Amaryllidaceae	*Haemanthus coccineus* L.	Poultice	Leaves	Topical	[27]
113	Asteraceae	*Haplocarpha scaposa* Harv.	Paste	Leaves	Topical, wash	[44]
114	Anacardiaceae	*Harpephyllum caffrum* Bernh.	Decoction Decoction	Bark Bark	Topical Oral, wash	[44] [8]
115	Asteraceae	*Helichrysum pedunculatum* Hillard & B.LBurtt	Not reported Juice	Leaves Leaves	Topical Topical	[47] [27]
116	Asteraceae	*Helichrysum appendiculatum* (L.f.) Less.	Poultice and infusion	Leaves	Topical	[27]
117	Asteraceae	*Helichrysum aureonitens* Sch.Bip	Infusion, lotion	Leaves	Topical, wash	[27]
118	Asteraceae	*Helichrysum odoratissimum* (L.) Sweet.	Infusion Poultice	Leaves Not reported	Topical Topical	[44] [43]
119	Asteraceae	*Helichrysum petiolare* Hilliard & B.L.Burtt	Decoction	Leaves	Oral, steam	[8]
120	Asteraceae	*Helichrysum nudifolium* (L.) Less.	Poultice, powder	Leaves, Twigs	Topical, steam	[8]
121	Malvaceae	*Hermannia cuneifolia* Jacq.	Poultice	Not reported	Wash	[36]
122	Malvaceae	*Hermannia depressa* N.E.Br	Not reported	Root	Topical	[34]
123	Apiaceae	*Heteromorpha arborescens* (Spreng.) Cham. & Schltdl.	Paste	Leaves	Topical	[35]
124	Hypoxidaceae	*Hypoxis hemerocallidea* Fisch., C.A.Mey. & Avé-Lall.	Not reported Not reported Juice, lotion, powder, infusion	Not reported Bulb Leaves and corms	Not reported Not reported Topical, wash	[29] [9] [27]
125	Hypoxidaceae	*Hypoxis rigidula* Baker	Not reported	Not reported	Not reported	[29]
126	Euphorbiaceae	*Jatropha curcas* L.	Infusion, Crushed	Roots	Topical, wash	[35]
127	Euphorbiaceae	*Jatropha zeyheri* Sond.	Paste	leaves	Topical	[35]
128	Juncaceae	*Juncus lomatophyllus* L.	Decoction	Leaves	Topical	[47]
129	Asphodelaceae	*Kniphofia drepanophylla* (Baker)	Powder, infusion	Rhizomes	Topical	[8]
130	Anacardiaceae	*Lannea schweinfurthii* var. *stuhlmannii* (Engl.) Kokwaro	Crushed	Leaves	Topical	[35]
131	Lamiaceae	*Leonotis leonurus* (L.) R.Br.	Infusion Not reported	Leaves Not reported	Oral Not reported	[8] [36]
132	Fabaceae	*Lessertia frutescens* (L.) Goldblatt & J.C.Manning subsp. *Frutescens = Sutherlandia frutescens* (L.) R. Br	Infusion Infusion	Leaves Leaves	Topical Topical	[8] [43]
133	Verbenaceae	*Lippia javanica* (Burm.f.) Spreng.	Poultice, maceration Infusion Not reported	Leaves Leaves Not reported	Topical Topical Not reported	[23] [44] [29]
134	Boraginaceae	*Lobostemon paniculatus* (Thunb.) H.Buek	Powder, poultice	Leaves	Topical	[43]
135	Lycopodiaceae	*Lycopodium clavatum* L.	Powder, decoction	Whole plant	Topical, oral	[34]
136	Euphorbiaceae	*Macaranga capensis* (Baill.) Sim	Paste, decoction	Bark	Topical, oral	[8]
137	Malvaceae	*Malva parviflora* L.var. parviflora	Paste Poultice	Leaves Not reported	Topical Not reported	[44] [36]
138	Melianthaceae	*Melianthus comosus* Vahl.	Decoction Not reported	Leaves Not reported	Wash Wash	[46] [36]
139	Melianthaceae	*Melianthus major* L.	Poultice	Foliage	Topical	[24]
140	Melianthaceae	*Melianthus pectinatus* Harv.	Poultice	Leaves	Topical	[43]
141	Lamiaceae	*Mentha longifolia* (L) L.	Poultice, lotion, infusion	Leaves	Topical	[27]
142	Hyacinthaceae	*Merwilla plumbea* (Lindl.) Speta	Powder, decoction, infusion	Bulbs	Topical, oral	[34]
143	Poaceae	*Miscanthus capensis* (Nees) Andersson	Decoction	Roots	Steam	[8]
144	Musaceae	*Musa acuminata* Colla	Not reported Not reported	Flowers, leaves Not reported	Not reported Not reported	[35] [29]
145	Musaceae	*Musa x paradisiaca* L.	Poultice	Leaves	Topical	[23]
146	Solanaceae	*Nicotiana glauca* Graham	Poultice Poultice	Leaves Not reported	Topical Topical	[43] [36]
147	Solanaceae	*Nicotiana tabacum L.*	Paste	Leaves	Topical	[44]
148	Ranunculaceae	*Nigella sativa* L.	Poultice	Whole plant	Oral, Topical	[33]
149	Scrophulariaceae	*Nemesia fruticans* (Thumb.) Benth.	Not reported	Not reported	Not reported	[36]
150	Cactaceae	*Opuntia vulgaris* Mill.	Poultice	Stems	Topical	[47]
151	Asteraceae	*Osteospermum calendulaceum* L.f.	Ointment	Not reported	Wash	[36]
152	Asteraceae	*Osteospermum herbaceum* L.f.	Poultice, infusion	Not reported	Topical	[28]
153	Anacardiaceae	*Ozoroa sphaerocarpa* R.Fern. & A.Fern.	Decoction, infusion	Whole plant	Not reported	[42]
154	Chrysobalanaceae	*Parinari curatellifolia* Planch. ex Benth.	Decoction, poultice	Leaves	Topical	[23]
155	Parmeliaceae	*Parmelia* species	Poultice Ointment	Not reported Not reported	Topical Topical	[43] [30]
156	Geraniaceae	*Pelargonium antidysentericum* (Eckl. & Zeyh.) Kostel.	Not reported	Not reported	Not reported	[30]
157	Geraniaceae	*Pelargonium grossularioides* (L.) L’Hér.	Powdered	Leaves	Topical	[36]
158	Geraniaceae	*Pelargonium luridum* (Andrews) Sweet	Not reported	Roots	Not reported	[9]
159	Geraniaceae	*Pelargonium peltatum* (L.) L’Hér.	Poultice	Leaves	Topical	[27]
160	Rubiaceae	*Pentanisia prunelloides* (Klotzsch ex Eckl. & Zeyh.) Walp.	Decoction Decoction	Root Root	Topical Wash	[34] [8]
161	Polygonaceae	*Persicaria lapathifolia* (L.) Delarbre	Not reported	Not reported	Not reported	[29]
162	Solanaceae	*Physalis angulata* L.	Paste	Leaves	Topical	[44]
163	Piperaceae	*Piper capense* L.f.	Maceration	Bark	Topical, Oral	[23]
164	Plantaginaceae	*Plantago lanceolata* L.	Decoction Poultice	Leaves Not reported	Topical Topical	[38] [36]
165	Polypodiaceae	*Polystichum pungens* (Kaulf.) C.Presl	Powder	Fronds	Topical	[27]
166	Didiereaceae	*Portulacaria afra* Jacq.	Poultice	Not reported	Not reported	[36]
167	Urticaceae	*Pouzolzia mixta* Solms	Powder	Roots	Topical	[35]
168	Anacardiaceae	*Protorhus longifolia* (Bernh.) Engl.	Decoction	Bark	Wash	[8]
169	Rosaceae	*Prunus persica* (L.) Batsch	Maceration	Bark	Topical	[23]
170	Rutaceae	*Ptaeroxylon obliquum* (Thunb.) Radlk.	Not reported	Not reported	Not reported	[29]
171	Icacinaceae	*Pyrenacantha kaurabassana* Baill.	Powder	Root tuber	Topical	[32]
172	Malvaceae	*Radyera urens* (L.f.) Bullock	Poultice	Leaves	Topical	[43]
173	Euphorbiaceae	*Ricinus communis* L.	Ointment, poultice Poultice Not reported Poultice	Seeds Roots and leaves Leaves Leaves	Topical Topical Not reported Topical	[43] [27] [41] [36]
174	Polygonaceae	*Rumex crispus* L.	Poultice	Leaves	Topical	[36]
175	Polygonaceae	*Rumex lanceolatus* Thunb.	Decoction	Roots, leaves	Wash	[8]
176	Celastraceae	*Salacia rehmannii* Schinz	Maceration	Bark	Topical	[23]
177	Asparagaceae	*Sansevieria cylindrica* Bojer ex Hook.	Not reported	Not reported	Not reported	[29]
178	Caprifoliaceae	*Scabiosa columbaria* L.	Ointment Powder	Roots Roots, leaves	Topical Topical	[27] [8]
179	Amaryllidaceae	*Scadoxus multiflorus* (Martyn) Raf. subsp. *katharinae*	Infusion	Bulb	Topical	[44]
180	Anacardiaceae	*Schinus molle* L.	Poultice Not reported	Leaves Foliage	Topical Topical	[43] [24]
181	Asparagaceae	*Scilla nervosa* (Burch.) Van der Merwe	Ointment	Bulb	Topical	[44]
182	Anacardiaceae	*Sclerocarya birrea* (A. Rich.) Hochst.	Burned Not reported	Stem Bark	Topical Not reported	[35] [15]
183	Anacardiaceae	*Searsia lancea* (L.f.) F.A. Barkley	Poultice, paste	Leaves	Topical	[23]
184	Asteraceae	*Senecio cinerascens* Aiton	Poultice	Leaves	Topical	[43]
185	Asteraceae	*Senecio deltoideus* Less.	Lotion	Leaves	Topical	[44]
186	Asteraceae	*Senecio speciosus* Willd.	Decoction, paste	Leaves, stems	Topical, steam	[8]
187	Fabaceae	*Senna obtusifolia* (L.) H.S.Irwin & Barneby	Poultice	Leaves	Topical, wash	[23]
188	Fabaceae	*Senna occidentalis* (L.) Link	Paste	Leaves	Topical	[35]
189	Malvaceae	*Sida cordifolia* L.	Burned ash	Roots	Topical	[35]
190	Solanaceae	*Solanum aculeastrum* Dunal	Not reported	Not reported	Not reported	[29]
191	Solanaceae	*Solanum incanum* L.	Paste Not reported	Roots Not reported	Topical Not reported	[8] [29]
192	Solanaceae	*Solanum lichtensteinii* Willd.	Not reported	Whole plant	Not reported	[9]
193	Solanaceae	*Solanum nigrum* L.	Infusion	Leaves	Wash	[27]
194	Solanaceae	*Solanum panduriforme* Drège ex Dunal	Juice	Fruit	Topical	[35]
195	Solanaceae	*Solanum tomentosum* L.	Not reported	Not reported	Not reported	[30]
196	Euphorbiaceae	*Spirostachys africana* Sond.	Infusion Not reported Powder (solution)	Bark Not reported Bark	Not reported Not reported Wash	[45] [29] [8]
197	Orobanchaceae	*Striga asiatica* (L) Kuntze	Maceration, paste Burned Burned	Roots Whole plant Whole plant	Topical Topical Topical	[23] [13] [35]
198	Loganiaceae	*Strychnos aculeata* Soler.	Maceration, lotion	Roots	Topical	[23]
199	Loganiaceae	*Strychnos decussata* (Pappe) Gilg	Not reported	Not reported	Not reported	[29]
200	Loganiaceae	*Strychnos henningsii* Gilg	Not reported	Not reported	Not reported	[29]
201	Myrtaceae	*Syzygium cordatum* Hochst. ex Krauss	Not reported Decoction	Bark Leaves, stems	Not reported Topical	[15] [45]
202	Apocynaceae	*Tabernaemontana elegans* Stapf	Maceration, lotion Not reported	Roots Latex (leaf)	Topical Topical	[23] [45]
203	Combretaceae	*Terminalia sericea* Burch. ex DC	Poultice, maceration	Leaves	Topical	[23]
204	Lamiaceae	*Tetradenia riparia* (Hochst.) Codd	Not reported	Not reported	Not reported	[29]
205	Santalaceae	*Thesium strictum* P.J.Bergius	Paste	Leaves	Topical	[44]
206	Commelinaceae	*Tradescantia pallida* (Rose) D.R. Hunt	Not reported	Not reported	Not reported	[29]
207	Meliaceae	*Trichilia emetica* Vahl	Poultice	Leaves	Topical	[23]
208	Alliaceae	*Tulbaghia alliacea* L.f.	Juice	Bulb	Topical	[8]
209	Crassulaceae	*Tylecodon wallichii* (Harv.) Toelken	Poultice	Leaves	Topical	[43]
210	Typhaceae	*Typha capensis* (Rohrb.) N.E.Br.	Infusion	Root and lower stem	Wash	[27]
211	Urticaceae	*Urtica urens* L.	Infusion	Leaves	Topical	[38]
212	Fabaceae	*Vachellia nilotica* subsp. *kraussiana* (Benth.) Kyal. & Boatwr.	Decoction	Roots	Not reported	[42]
213	Rubiaceae	*Vangueria infausta* Burch.	Decoction	Roots	Not reported	[42]
214	Rutaceae	*Vepris lanceolata* (Lam) G.Don	Not reported	Not reported	Not reported	[29]
215	Asteraceae	*Vernonia oligocephala*	Infusion, lotion	Leaves and stems	Topical	[27]
216	Solanaceae	*Withania somnifera* (L.) Dunal	Paste Poultice Poultice	Leaves, roots Leaves Not reported	Topical Topical Not reported	[8] [27] [36]
217	Olacaceae	*Ximenia caffra* Sond.	Maceration, poultice	Roots	Topical	[23]
218	Apocynaceae	*Xysmalobium undulatum* (L.) Aiton f.	Powder	Roots	Topical	[34]
219	Araceae	*Zantedeschia aethiopica* (L.) Spreng.	Poultice, heated Powder Poultice	Leaves Rhizome Leaves	Topical Topical Topical	[27] [44] [36]
220	Rutaceae	*Zanthoxylum davyi* (I.Verd.) P.G.Waterman	Crushed	Roots, leaves	Topical	[35]
221	Poaceae	*Zea mays L.*	Paste	Leaves	Topical	[44]
222	Rhamnaceae	*Ziziphus mucronata* Willd. subsp. *mucronata*	Poultice	Leaves	Topical	[23]

## Data Availability

No new research data was created. All data was retrieved from published articles available to the public from various databases outlined in the Section 3 of the paper.
